# Treating cellulitis promptly with compression therapy reduces C‐reactive protein‐levels and symptoms – a randomized‐controlled trial

**DOI:** 10.1111/ddg.15829

**Published:** 2025-08-11

**Authors:** Sören Dräger, Charlotte Kiehne, Greta Zinser, Birgit Kahle

**Affiliations:** ^1^ Department of Dermatology Allergology and Venereology University Medical Center Schleswig‐Holstein (UKSH), Campus Lübeck Lübeck Germany; ^2^ Department of Dermatology University Hospital Duesseldorf Duesseldorf Germany

**Keywords:** cellulitis, compression therapy, medical adaptive compression wraps

## Abstract

**Background and Objectives:**

Cellulitis is an acute bacterial infection of the skin. Initial treatment primarily consists of systemic antibiotic therapy. Compression therapy is subsequently introduced to reduce edema. However, the optimal timing for initiating compression therapy remains a subject of debate, as early application is thought to potentially exacerbate the infection.

**Patients and Methods:**

This study was designed as a prospective, randomized controlled trial. Patients admitted for treatment of lower leg cellulitis were recruited and randomly assigned in a 1:1 ratio. In addition to standard therapy, the intervention group received compression therapy one day after initiation of antibiotic treatment, using medical adaptive compression wraps. C‐reactive protein (CRP) levels, reduction in erythema, and patient‐reported symptoms were recorded.

**Results:**

A total of 34 patients were included in the analysis. Early application of medical adaptive compression wraps alleviated symptoms without causing complications. In patients with initial CRP levels above 50 mg/dl at admission, CRP reduction occurred more rapidly.

**Conclusions:**

Our data suggest that the early application of medical adaptive compression wraps within 24 hours of initiating antibiotic treatment alleviates symptoms, supports recovery, and does not lead to worsening of inflammation.

## INTRODUCTION

Cellulitis is a rapidly developing bacterial infection that primarily affects the skin, subcutaneous tissue, and dermal lymphatics. It is most commonly caused by β‐hemolytic streptococci, usually group A, or *Staphylococcus aureus*.^1^ In many cases, minor skin barrier defects, often caused by fungal maceration in the interdigital spaces of the feet or an ulceration, serve as entry points for these pathogens. The risk for a primary episode of cellulitis is increased by chronic venous insufficiency, lymphedema, tinea pedis, and others.^2^ The diagnosis of cellulitis is based on its characteristic clinical presentation, which typically includes asymmetrical, well‐defined erythema, localized heat, and swelling. In many cases, these symptoms are accompanied by fever. The lower legs, arms, and trunk are the most frequently affected body regions.^3^ Laboratory findings typically include unspecific elevated markers of inflammation, such as leukocytosis and increased C‐reactive protein (CRP) levels. Direct pathogen detection is generally unsuccessful and, therefore, plays a minor role in the diagnostic process. Complicating localized erosions, blisters or hemorrhagic lesions can occur.^4^ Nonetheless, cellulitis may be complicated by bacteremia or sepsis, conditions which are potentially life‐threatening. A common secondary complication that occurs as a result of cellulitis is lymphoedema, which in turn results in an increased risk of recurrent cellulitis.^5^, ^6^ The mainstay of treatment for cellulitis is antibiotic therapy, with penicillin typically recommended as the first‐line agent in Germany.^7^ Supportive measures include physical rest, cooling compresses, pain management and addressing the point of entry.

Additionally, compression therapy can be employed. It enhances skin perfusion, promotes lymphatic drainage, effectively reduces edema, and is commonly used in the management of venous and lymphatic disorders.^8^, ^9^, ^10^ Before initiation, routine clinical evaluation of the peripheral arterial circulation should be performed – for example, by palpating the A. dorsalis pedis or posterior tibial artery – especially in patients at risk, such as those with peripheral arterial occlusive disease, diabetes, or peripheral neuropathy. The main contraindications for compression therapy include an ankle–brachial index (ABI) below 0.5, systolic ankle pressure below 60 mmHg, septic phlebitis, phlegmasia coerulea dolens, and decompensated heart failure.^10^ There is ongoing debate about the optimal initiation time point for compression therapy.^11^ Previously, compression therapy was initiated several days after the start of antibiotic treatment, based on the assumption that applying compression during the acute inflammatory phase could exacerbate the infection or even trigger sepsis. However, recent studies have shown that compression therapy does not negatively affect the course of cutaneous bacterial infections. On the contrary, it appears to facilitate recovery.^11^ Secondary prevention of recurrent cellulitis using compression therapy is well‐established and cost‐effective.^12^, ^13^ There are various compression systems, including medical adaptive compression wraps (MAK). The advantage of MAK is their ability to dynamically adjust to changing limb circumferences, particularly during the decongestion phase. Moreover, their ease of use and the patient's ability to apply them independently are additional advantages that enhance compliance and promote regular use.

In summary, there is currently a lack of studies examining the optimal timing for initiating compression therapy. To address this question, the present study was designed to evaluate whether early initiation of compression therapy – applying MAK within 24 hours of starting antibiotic treatment – leads to a more rapid reduction in CRP levels, a decrease in erythema size, and an improvement in symptoms.

## METHODS

**FIGURE 1:**
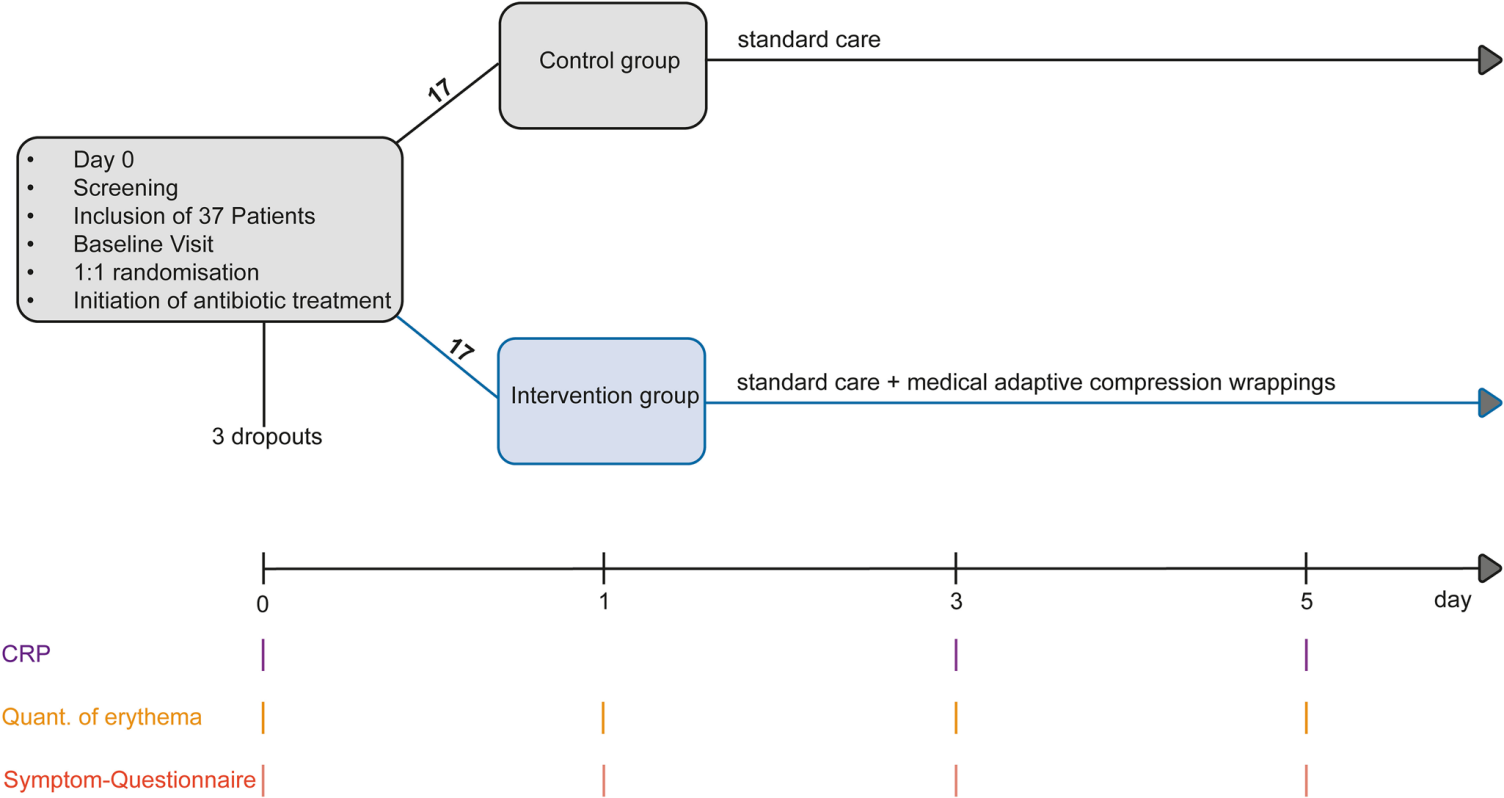
Schematic representation of the study. On the day of admission (day 0), antibiotic treatment was initiated. Patients were then screened and, after giving informed consent, the baseline visit was performed (C‐reactive protein [CRP] levels, erythema, and symptom questionnaire). From then on, both the control group and the intervention group received standard care. On day 1, patients in the intervention group additionally received individually fitted medical adaptive compression wraps and were educated in their use. Erythema quantification and the symptom questionnaire were reassessed on days 1, 3, and 5, while CRP levels were measured on days 3 and 5. After day 5, allocated treatment was continued, but no further assessments were conducted for this study.

This trial was conducted as a prospective, randomized controlled study, approved by the local ethics committee of the University of Luebeck (AZ 21–043) and in accordance with the Helsinki Declaration of 1975. Following informed consent, patients aged 18 and above, who were admitted for the treatment of cellulitis of the lower legs, were enrolled in the study. All patients were treated at the Department of Dermatology, Allergology, and Venereology at the University Medical Center Schleswig‐Holstein (UKSH), Campus Lübeck, between October 2021 and March 2024. Exclusion criteria included the presence of phlegmon, decompensated heart failure, and inability to independently apply the MAK. Participants were randomly assigned in a 1:1 ratio to two groups: The control group received standard therapy, consisting of intravenous antibiotics, antiseptic compresses, and restricted bed rest. The MAK group received the same standard therapy as the control group, with the addition of compression therapy using medical adaptive compression, initiated 24 hours after the start of antibiotic treatment (Figure 1). A 24‐hour interval was selected as a defined time window to allow the antibiotic therapy to take effect before initiating compression therapy. In this group, antiseptic wrappings were applied for 30 minutes twice daily prior to the application of MAK. Treatment allocation was performed using sequentially numbered, sealed envelopes, which were opened after the patient had provided informed consent. The random allocation sequence was generated by the Institute for Medical Biometry and Statistics at the University of Lübeck, Germany. The assigned treatment was continued until discharge. Medical treatment and clinical decision‐making were carried out by physicians who were not involved in the conduct of the study.

The primary endpoint of the study was the reduction in CRP levels, which was assessed on days 0, 3, and 5. Secondary endpoints were the change of the area of erythema to baseline, and the reported symptoms. The area of erythema was quantified on days 0, 1, 3 and 5 by placing a defined transparent film on the affected area and marking the boundaries of the erythema onto the film. The film was then trimmed accordingly and weighed. The absolute values in grams were recorded and the relative change to the baseline value calculated. The symptoms were assessed on days 0, 1, 3, and 5 using a paper‐based modified symptom questionnaire, which included seven items, each rated on a scale from 0 to 10. The evaluated items were the following: *(1.)* How strongly do you feel impaired by your illness? *(2.)* How strong is your pain? *(3.)* How much discomfort causes the swelling of your leg? *(4.)* How much discomfort causes the swelling of your foot? *(5.)* Do you have pain when walking? *(6.)* Do you have pain when sitting? *(7.)* How discomforting is the compression? Item 7 applied exclusively to the MAK group and was therefore evaluated separately. In addition, data on medication, medical history, and length of hospital stay were extracted from the medical records.

Sample size calculation was performed with an alpha level of 5%, a power of 80%, and an assumed treatment effect size of 20%. Statistical analysis was conducted using GraphPad Prism version 10.0 (GraphPad Software, Inc.), RStudio (Posit Software PBC), and Microsoft Excel version 16.87 (Microsoft). A p value of < 0.05 was considered statistically significant. Significance was assessed using the unpaired Student's *t*‐test. A subgroup analysis was conducted for patients with an initial CRP level > 50 mg/dl at admission.

## RESULTS

### Epidemiology of the study population

The study included 37 patients. Three patients were excluded from the analysis: two (one from each group) because no further data beyond day 1 were collected, and one patient discontinued the study after day 1 due to intolerance of compression therapy. Baseline characteristics of the remaining 34 patients, split equally between the control (n  =  17) and intervention (n  =  17) groups, are summarized in Table 1. In the control group, 35.3 % of patients were female and 64.7% male, compared to 29.4% female and 70.6% male in the intervention group. The mean age was 59.0 ± 23.8 years in the control group and 57.2 ± 17.1 years in the intervention group, with an overall mean of 58.1 ± 20.4 years. The average body mass index (BMI) was 28.98 kg/m^2^ in the control group and 33.30 kg/m^2^ in the intervention group, indicating a higher BMI in the intervention group.

**TABLE 1:** Epidemiology of the study population. Age and sexwere comparable between groups, while body mass index (BMI) was higher in the intervention group. Comorbidities were categorized by organ system, including cardiovascular, kidney, autoimmune, and respiratory disorders, as well as metabolic, neurologic, and psychiatric conditions. In addition, a history of cancer and previous cellulitis episodes was recorded. Frequencies are presented as absolute numbers and corresponding percentages.

Group	Control		Intervention (MAK)		Total	
	*Number*	*Percentage*	*Number*	*Percentage*	*Number*	*Percentage*
Patients per group	17	50.00%	17	50.00%	34	100.00%
Females	6	35.29%	5	29.41%	11	32.35%
Males	11	64.71%	12	70.59%	23	67.65%
Age (mean)	59.00		57.16		58.08	
Age (standard deviation)	23.82		17.10		20.44	
BMI (kg/m^2^)	28.98		33.30		31.14	
Cardiovascular diseases	13	76.47%	9	52.94%	22	64.71%
Kidney diseases	2	11.76%	3	17.65%	5	14.71%
Respiratory diseases	2	11.76%	4	23.53%	6	17.65%
Metabolic disorders	3	17.65%	3	17.65%	6	17.65%
Diabetes mellitus	5	29.41%	2	11.76%	7	20.59%
Autoimmune diseases	2	11.76%	1	5.88%	3	8.82%
Cancer	4	23.53%	2	11.76%	6	17.65%
Neurological disorders	2	11.76%	0	0.00%	2	5.88%
Psychiatric disorders	1	5.88%	1	5.88%	2	5.88%
Previous cellulitis (any location)	1	5.88%	6	35.29%	7	20.59%

*Abbr*.: MAK, medical adaptive compression wrappings

With regard to medical history, cardiovascular diseases were the most common, affecting 76.5% of patients in the control group and 52.9% in the intervention group. Kidney diseases were reported in 11.8% of control group patients and 17.7% of those in the intervention group. Respiratory diseases affected 11.8% of patients in the control group and 23.5% of patients in the intervention group, while metabolic disorders were seen in 17.7% of both groups. Diabetes mellitus was more common in the control group (control: 29.4%, intervention: 11.8%). Other conditions included cancer (control: 23.5%, intervention: 11.8%), autoimmune diseases (control: 11.8%, intervention: 5.9%), and neurological disorders (control: 11.8%, intervention: 0%). Psychiatric disorders were equally distributed (control: 5.9%, intervention: 5.9%), while a history of previous cellulitis was more prevalent in the intervention group (control: 5.9%, intervention: 35.3%).

### Prompt application of MAK alleviates symptoms

**FIGURE 2:**
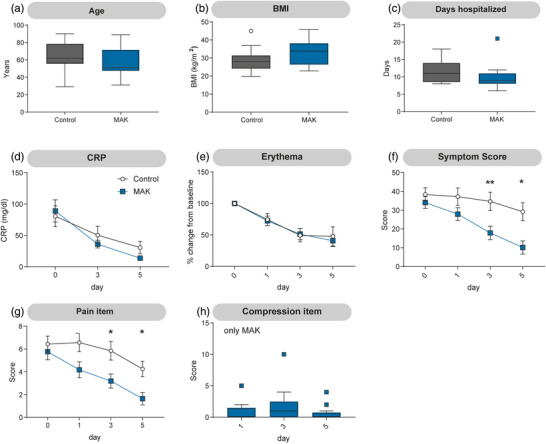
Application of MAK within 24 hours alleviates symptoms. (a) Age, (b) body mass index (BMI), (c) days hospitalized, (d) levels of C‐reactive protein (CRP), and (e) change in erythema from baseline were comparable between patients receiving MAK and the control group. (f) The symptom score, based on a questionnaire with six items, was significantly reduced from day 3 onward in patients receiving MAK. (g) This reduction was also observed for the pain item in the MAK group. (h) One patient reported discomfort from the MAK on day 3; however, for all other patients, the compression‐related discomfort score remained low throughout the study. (a–c): Tukey boxplot; d–h: mean ± SEM; n  =  17; a–g: unpaired Student's *t*‐test; *p < 0.05, **p < 0.005.

Patients who received treatment with MAK within 24 hours after initiation of antibiotic therapy reported a reduced symptom score (Figure 2f). Both groups started with a symptom score of 38.4 (control) and 34.1 (MAK), respectively. On day 3, patients in the control group reported a mean symptom score of 34.7, whereas MAK treatment led to a reduction to 17.9. This difference persisted on day 5, with a symptom score of 29.2 (control) and 10.1 (MAK). A comparable difference was also observed when evaluating the pain item of the questionnaire separately (Figure 2g).

No significant difference in hospitalization duration was found between groups. The control group had a mean hospital stay of 11.4 days, while the MAK group averaged 9.95 days (Figure 2c). However, the MAK group included one outlier (21 days due to DRESS syndrome at the end of treatment). When this outlier was excluded from analysis, the mean hospitalization duration changed slightly (11.4 vs. 9.3 days), and the difference became statistically significant (unpaired *t*‐test, p  =   0.024). One patient reported high discomfort due to compression therapy (Figure 2h). No effect of MAK treatment was observed on CRP levels (Figure 2d) or change in erythema (Figure 2e). The need for second‐ or third‐line antibiotic treatment was not more frequent in the MAK group (online supplemental Figure 1a). Pain medication use was comparable between groups (online supplemental Figure 1b).

### Subanalysis of patients with initial CRP values above 50 mg/dl reveals accelerated CRP reduction by day 5

**FIGURE 3:**
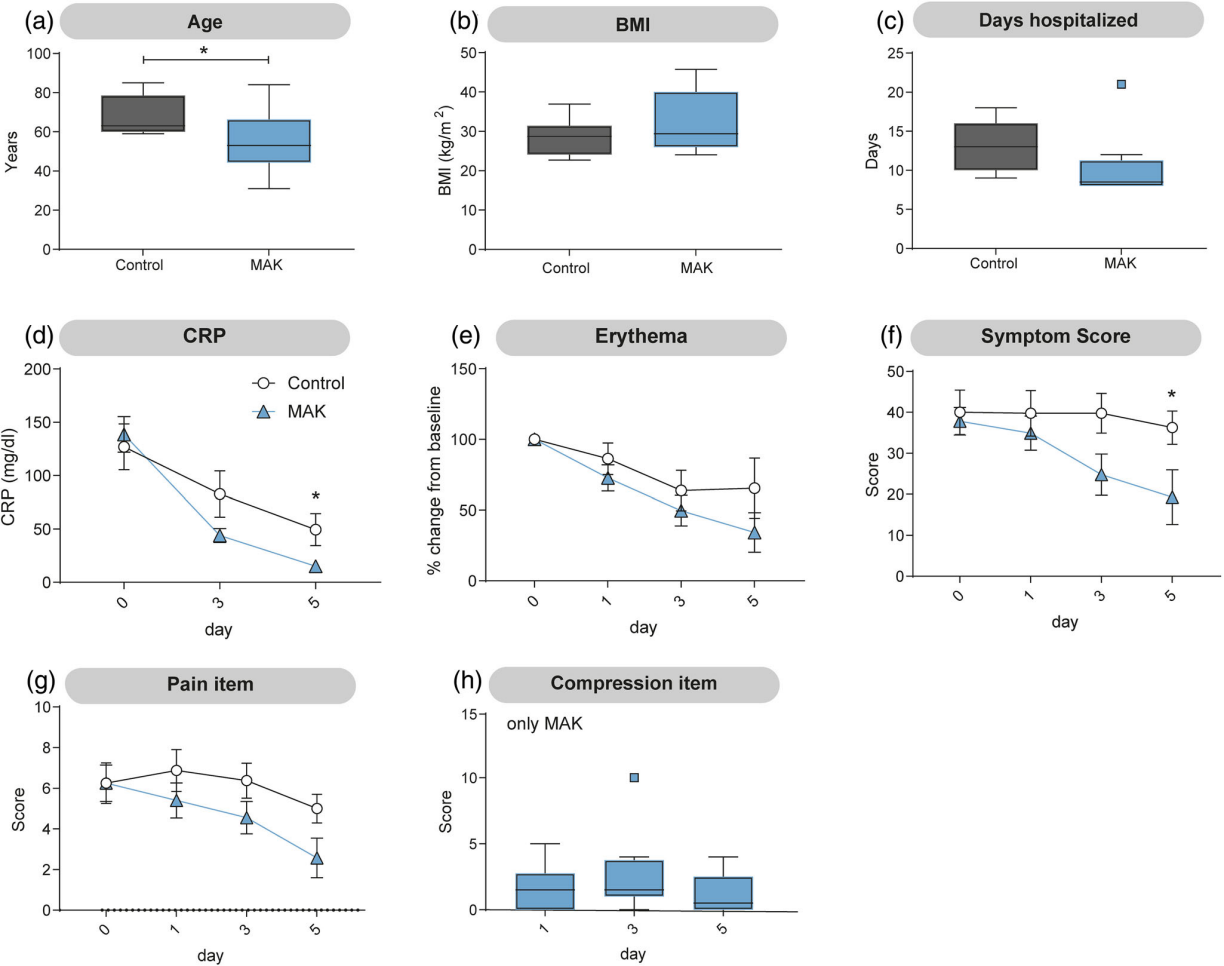
Subanalysis of patients with initial CRP values above 50 mg/dl reveals accelerated CRP reduction by day 5. (a) Age was significantly lower in the group receiving treatment with MAK. (b) Body mass index (BMI) and (c) days hospitalized were comparable between both groups. (d) Levels of C‐reactive protein (CRP) were significantly lower by day 5 in patients receiving MAK therapy. (e) Change in erythema from baseline was comparable between patients receiving MAK and the control group. (f) The questionnaire‐based symptom score was significantly reduced on day 5 in patients receiving MAK, while (g) no reduction in the pain item was noted. (h) Except for one patient, the compression‐related item remained low throughout the study. (a–c): Tukey boxplot; d–h: mean ± SEM; n  =  9; (a–g): unpaired Student's *t*‐test; *p < 0.05, **p < 0.005.

Since the inclusion criteria did not specify a minimum CRP level, patient values varied widely. Therefore, a subgroup analysis was conducted including only patients with initial CRP values > 50 mg/dl, comprising nine patients in each group (Figure 3). Unexpectedly, a significant age difference was observed between these groups, with a mean age of 68 years in the control group and 54.4 years in the MAK group (Figure 3a). BMI was comparable between groups (Figure 3b). The trend toward reduced hospitalization duration remained evident and would have reached statistical significance if the outlier had been excluded from the analysis (Figure 3c). Since inclusion in this analysis was based on CRP levels, the initial mean CRP increased from 80.6 to 126.9 mg/dl in the control group and from 89.0 to 138.5 mg/dl in the MAK group (Figure 2d). Interestingly, in this subgroup, CRP levels decreased more rapidly by day 5 in the MAK group. In the control group, CRP remained relatively high on day 5 with a mean of 49.3 mg/dl, whereas in the MAK group it was reduced to 15.0 mg/dl. Symptom scores differed significantly between groups on day 5; however, the previously observed difference in the pain item was no longer evident (Figure 3e,f).

### No bacteremia or sepsis associated with MAK‐treatment

As previously mentioned, one patient in the MAK group developed a drug eruption with systemic symptoms (DRESS syndrome) during the course of treatment, which led to prolonged hospitalization. In the control group, one patient developed a drug eruption and an abscess below the metatarsophalangeal joint that required surgical intervention. Another patient who had initially received MAK discontinued the study on day 2. On day 7, he developed a phlegmon, requiring escalation of antibiotic treatment to piperacillin/tazobactam.

## DISCUSSION

This study investigated whether early initiation of compression therapy – specifically, the application of MAK within 24 hours after the start of antibiotic treatment – accelerates the reduction of CRP levels, decreases erythema size, and alleviates symptoms. To this end, a total of 37 patients were recruited into two cohorts: an intervention group (standard care plus MAK) and a control group (standard care only). MAK were selected due to key advantages, including their adaptability to the decreasing circumference of the lower leg during treatment, ease of application and removal, and suitability for patient‐friendly self‐management. These features are particularly crucial during the acute inflammatory phase of cellulitis, where pain, discomfort, and leg swelling are the primary symptoms. We therefore evaluated the impact of MAK on the symptom score, which was significantly lower in patients receiving MAK therapy. Notably, the pain item score was reduced, potentially enhancing therapy adherence. MAK therapy was not well tolerated by one patient, as reflected by a high score in the compression item. In summary, MAK treatment is a feasible and well‐tolerated option for acute cellulitis in patients without contraindications for compression therapy. Before initiation, clinical examination of the peripheral arterial vasculature should be performed.

Overall, a comparable reduction in CRP levels was observed. However, initial CRP values varied considerably, as no minimum level was defined in the inclusion criteria. Therefore, a subgroup analysis was conducted including only patients with an initial CRP level > 50 mg/dl. This analysis revealed that CRP levels were lower in the MAK group by day 5. Since the aim of this study was to evaluate the effect of MAK during the acute phase, data collection was limited to the first 5 days. The effect of the MAK might have been even more pronounced at later time points. The values obtained in our study are comparable to those reported by Eder et al., who observed an initial mean CRP level of 124.00 mg/dl and a discharge value of 30.89 mg/dl. However, the study by Eder et al. did not include a control group.^11^

Regarding complications associated with the application of MAK in acute cellulitis, no differences were observed between the groups. The concern that compression during the acute phase may exacerbate the condition or trigger sepsis remains common. However, in our study, none of the patients developed complications such as bacteremia or sepsis, which is consistent with previous findings.^11^ Nevertheless, due to the small sample size, larger cohort studies are necessary to validate these findings.

Our data suggests that the prompt application of medical adaptive compression wraps within 24 hours after the initiation of antibiotic treatment alleviates symptoms, supports recovery, and does not provoke a deterioration of inflammation.

### Limitations

Our data are based on a sample size of 34 patients, which limits the generalizability of the findings. In addition, the study is constrained by its prospective controlled design without follow‐up. As data were collected exclusively during the inpatient stay, only primary complications could be assessed. Subsequent events, as well as medium‐ and long‐term outcomes that may have occurred during the further course of treatment, were therefore not captured. Nevertheless, the collected patient data are generally in line with current literature. However, the findings should be validated in studies with larger sample sizes and extended follow‐up periods.

## CONFLICT OF INTEREST STATEMENT

G.Z. received a research award from Julius Zorn GmbH. All other authors declare no conflicts of interest.

## Supporting information



Supplementary information

## References

[1] Raff AB , Kroshinsky D . Cellulitis: A Review. JAMA. 2016;316(3):325. 27434444 10.1001/jama.2016.8825

[2] Cannon J , Rajakaruna G , Dyer J , et al. Severe lower limb cellulitis: defining the epidemiology and risk factors for primary episodes in a population‐based case‐control study. Clin Microbiol Infect. 2018;24(10):1089‐1094. 29427797 10.1016/j.cmi.2018.01.024

[3] Ren Z , Silverberg JI . Burden, risk factors, and infectious complications of cellulitis and erysipelas in US adults and children in the emergency department setting. J Am Acad Dermatol. 2021;84(5):1496‐503. 33238162 10.1016/j.jaad.2020.11.021

[4] Perelló‐Alzamora MR , Santos‐Duran JC , Sánchez‐Barba M , et al. Clinical and epidemiological characteristics of adult patients hospitalized for erysipelas and cellulitis. Eur J Clin Microbiol Infect Dis. 2012;31(9):2147‐152. 22298240 10.1007/s10096-012-1549-2

[5] Cannon J , Dyer J , Carapetis J , Manning L . Epidemiology and risk factors for recurrent severe lower limb cellulitis: a longitudinal cohort study. Clin Microbiol Infect. 2018;24(10):1084‐1088. 29427799 10.1016/j.cmi.2018.01.023

[6] Pavlotsky F , Amrani S , Trau H . Recurrent erysipelas: risk factors: Risikofaktoren für Rezidiverysipele. J Dtsch Dermatol Ges. 2004;2(2):89‐95. 16279242 10.1046/j.1439-0353.2004.03028.x

[7] Sunderkötter C , Becker K , Eckmann C , et al. S2k guidelines for skin and soft tissue infections Excerpts from the S2k guidelines for “calculated initial parenteral treatment of bacterial infections in adults ‐ update 2018”. J Dtsch Dermatol Ges. 2019;17(3):345‐369. 10.1111/ddg.13790 30920735

[8] Klyscz T , Galler S , Steins A , et al. Einfluß einer Kompressionstherapie auf die Mikrozirkulation der Haut bei Patienten mit chronischer Veneninsuffizienz (CVI). Hautarzt. 1997;48(11):806‐811. 9518241 10.1007/s001050050664

[9] Dissemond J , Eder S , Läuchli S , et al. [Compression therapy for inflammatory dermatoses of the legs]. Dtsch Med Wochenschr. 2024;149(3):106‐112. 38262405 10.1055/a-2197-6197

[10] Rabe E , Földi E , Gerlach H , et al. Medizinische Kompressionstherapie der Extremitäten mit medizinischem Kompressionsstrumpf (MKS), phlebologischem Kompressionsverband (PKV) und medizinischen adaptiven Kompressionssystemen (MAK): S2k‐Leitlinie der Deutschen Gesellschaft für Phlebologie (DGP) in Kooperation mit folgenden Fachgesellschaften: DDG, DGA, DGG, GDL, DGL, BVP. Hautarzt. 2021;72(2):137‐152. 33301064 10.1007/s00105-020-04734-9

[11] Eder S , Stücker M , Läuchli S , Dissemond J . Ist die Kompressionstherapie bei Erysipel des Unterschenkels kontraindiziert?: Resultate einer retrospektiven Analyse. Hautarzt. 2021;72(1):34‐41. 32930854 10.1007/s00105-020-04682-4

[12] Webb E , Bissett B , Neeman T , et al. Compression Therapy Is Cost‐Saving in the Prevention of Lower Limb Recurrent Cellulitis in Patients with Chronic Edema. Lymphat Res Biol. 2023;21(2):160‐168. 35997601 10.1089/lrb.2022.0029 PMC10125391

[13] Webb E , Neeman T , Bowden FJ , et al. Compression Therapy to Prevent Recurrent Cellulitis of the Leg. N Engl J Med. 2020383(7):630‐639. 10.1056/NEJMoa1917197 32786188

