# Supplementary figures and images for: Treating cellulitis promptly with compression therapy reduces C‐reactive protein‐levels and symptoms – a randomized‐controlled trial
Source: J Dtsch Dermatol Ges. 2025 Aug 11;23(10):1274–80. doi: 10.1111/ddg.15829 (PMC12548327; doi:10.1111/ddg.15829)

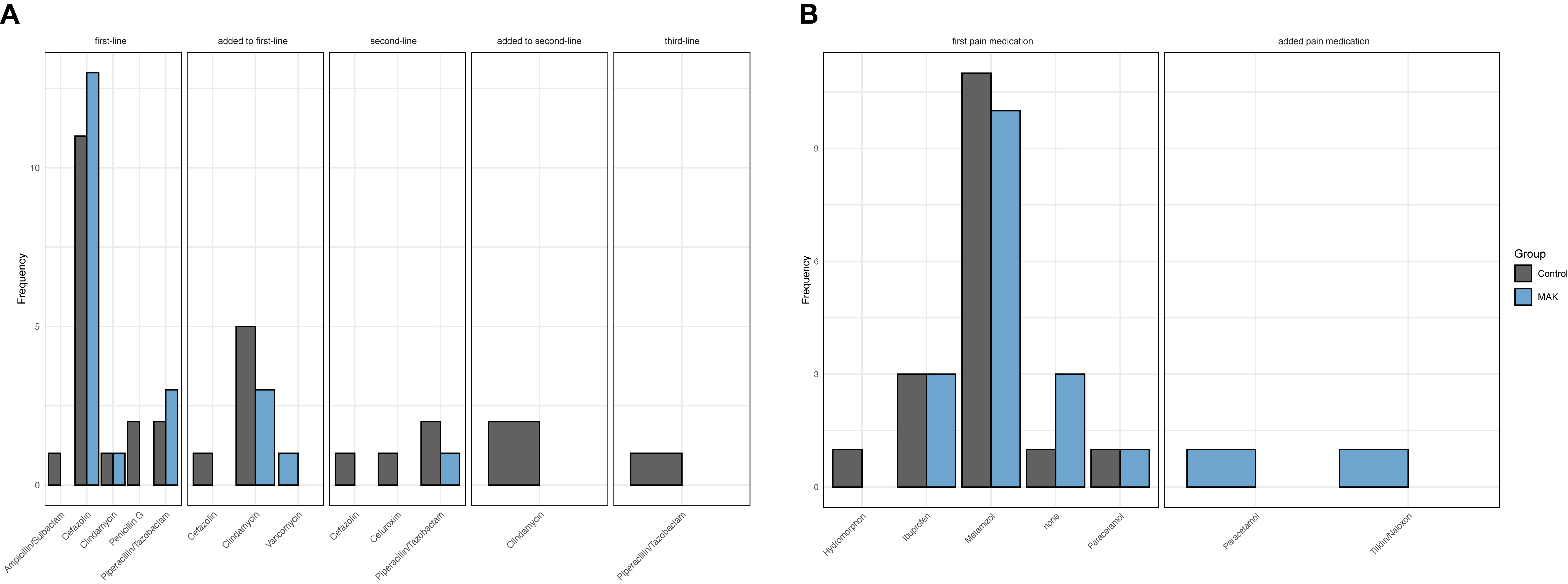

Supplement: Supplementary file 1 — Supplementary information [file DDG-23-1274-s001.jpg]
